# The Possible Unveiling of Myasthenia Gravis via Allergy Shots

**DOI:** 10.7759/cureus.91927

**Published:** 2025-09-09

**Authors:** Ami Thakor Philipp, Lorraine Anderson

**Affiliations:** 1 Allergy and Immunology, University of California Los Angeles, Los Angeles, USA

**Keywords:** allergic rhinoconjunctivitis, allergy immunotherapy, allergy shots, anaphylaxis, myasthenia gravis

## Abstract

This case report discusses subcutaneous allergen immunotherapy (SCIT) for allergic rhinoconjunctivitis and explores whether myasthenia gravis symptoms developed in a patient due to subcutaneous allergen immunotherapy treatment. SCIT is known to induce immune tolerance through the generation of allergen-specific regulatory T and B cells, increased allergen-specific IgG4, and suppression of effector cell responses, resulting in sustained clinical benefit for allergic disease. Although SCIT is considered safe and is not associated with an increased risk of autoimmune disease in large population studies, rare case reports have raised concerns about its potential to trigger autoimmune conditions. The most common triggers for autoimmune diseases, including myasthenia gravis, remain unclear, but allergic diseases themselves may confer an increased risk. This case report highlights the need for further research into the immunological mechanisms and potential triggers of autoimmunity in the context of allergen immunotherapy.

## Introduction

Myasthenia gravis is a neurologic autoimmune condition that can present in a multitude of ways. Clinical features include ocular symptoms like diplopia or ptosis, bulbar symptoms (e.g., dysphagia, dysarthria, dysphonia), limb weakness, respiratory symptoms, and fatigue. The antibody-mediated attack on acetylcholine receptors in the neuromuscular junction causes these symptoms [1]. Myasthenic crisis is something that can be provoked by certain medications, infections and inflammation. It is an acute, life-threatening exacerbation of myasthenia gravis that can lead to respiratory failure. While there aren't any clear indicators in the literature that subcutaneous allergen immunotherapy (SCIT) can provoke myasthenia gravis, one can consider SCIT an immune modulator that may provoke symptoms of myasthenic crisis in a patient with underlying myasthenia gravis.

## Case presentation

The patient is a 65-year-old man who presents with rhinoconjunctivitis. He states that he has a long history of allergic rhinitis and was previously on allergen immunotherapy about 30 years ago. Allergen immunotherapy improved his symptoms, but they have recently returned. He has noticed tearing and “stickiness” around the eyes, and sometimes his right eye droops and feels itchy. He has not noted any weakness or neurological symptoms. 

Percutaneous testing was done for environmental allergens during his visit. He was found to be highly allergic to trees, grasses, and weeds. SCIT was offered, and he was placed on it shortly thereafter. His symptoms initially improved with SCIT, but about seven months into the regimen, he had presumed anaphylaxis after an allergen injection. He was treated with epinephrine intramuscularly and saw improvement. Upon his follow-up visit a few months later, he mentioned dysphagia and jaw pain. He also noticed some fatigue, possibly related to SCIT or his allergy symptoms. At that time, myasthenia gravis diagnosis was considered due to his symptoms, and testing was done. The acetylcholine receptor-blocking antibody was 47% (reference range < 15%). He was referred to neurology and placed on treatment with pyridostigmine 60mg orally three times a day. He had complete resolution of his symptoms of ptosis, dysphagia, jaw pain, and fatigue. In addition, his allergic rhinoconjunctivitis symptoms resolved as he continued SCIT. He has continued SCIT with the support of his neurologist for his allergic rhinoconjunctivitis. He has seen improvement but has noticed some mild myasthenia gravis symptoms on days of his injections. He has not had anaphylaxis again or any systemic symptoms after SCIT.

**Figure 1:**
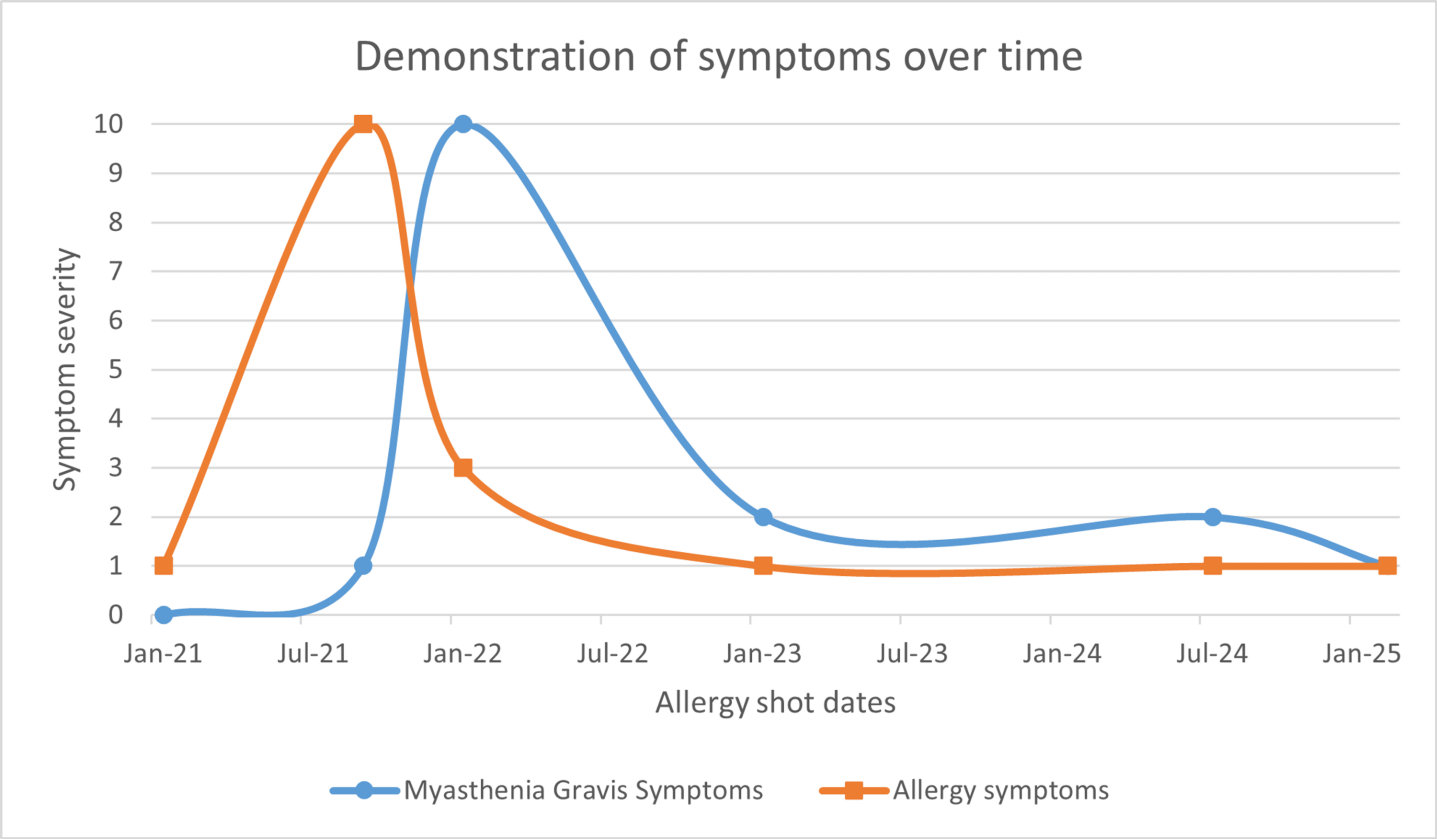
Demonstration of symptoms over time This figure demonstrates the patient's reported symptoms in the period of time while on subcutaneous allergen immunotherapy (SCIT). The patient would report any symptoms on a scale of 0-10, with 0 being asymptomatic and 10 being high severity of symptoms. Allergy symptoms included nasal congestion, runny nose, sneezing and cough. Myasthenia gravis symptoms included ptosis, generalized weakness, and fatigue. By the end of SCIT therapy, the patient was asymptomatic.

Figure 1 shows how this patient’s symptoms evolved over time. His self-reported symptoms are noted on a scale of 0-10, 10 being the worst. This only demonstrates his reported symptoms and is not related to his physical exam findings. Overall, his physical exam findings did not change significantly during this time period. 

## Discussion

Historically, allergen immunotherapy has been on the treatment algorithm for allergic rhinitis, allergic asthma, and allergic conjunctivitis. It is very effective for these conditions [2]. Few studies have examined whether there is any relationship between allergic diseases and myasthenia gravis. Some studies have shown a link between allergic diseases and developing myasthenia gravis later in life. However, there is no direct evidence of SCIT causing myasthenia gravis in the literature [3]. Because of the autoimmune nature of myasthenia gravis, it is theoretically possible that any immune-modulating treatment could influence the disease course. SCIT is known to induce immune tolerance through the generation of allergen-specific regulatory T and B cells, increased allergen-specific IgG4, and suppression of effector cell responses, resulting in sustained clinical benefit for allergic disease [4]. This immune change should not trigger autoinflammation like what would be found in myasthenia gravis. In addition, no randomized control trials to date have suggested a link between SCIT and the development of autoimmune conditions, just some case reports [5]. The cases reported were primarily patients who developed vasculitis after starting allergen immunotherapy [6]. However, it is important to continue to monitor for any potential untoward side effects or symptoms from SCIT, especially in patients with underlying medical conditions. A study done in 2016 assessed this by web-based surveys of allergists/immunologists. It determined certain underlying conditions that are associated with risk in SCIT [7]. Autoimmune conditions, including myasthenia gravis, were not among them. 

## Conclusions

This case shows symptoms of classic allergic rhinoconjunctivitis in a person seen in the context of underlying myasthenia gravis. Although SCIT did help the patient with his nasal and ocular symptoms, it may have triggered myasthenia gravis symptoms or caused him to present symptoms earlier. He ultimately found a balance between SCIT and myasthenia gravis treatment and had complete resolution of all symptoms from both conditions. He was able to do this by titrating his pyridostigmine dose with neurology as well as continuing on his SCIT treatment. The patient is grateful for the combination of treatments given to him because his symptoms resolved. In conclusion, it is acceptable to continue SCIT in patients with myasthenia gravis if they are tolerating the therapy. In addition, it may help resolve inflammation and other symptoms of both myasthenia gravis and allergic rhinoconjunctivitis.
